# Complete Resolution of Cutaneous Leishmaniasis With a Novel Topical Combination Therapy: A Case Series

**DOI:** 10.1002/ccr3.71211

**Published:** 2025-10-07

**Authors:** Hoda Haghshenas, Aref Ghanaatpisheh, Mohammad Darayesh, Elaheh Entezar Almahdi

**Affiliations:** ^1^ Faculty of Medicine Jahrom University of Medical Sciences (JUMS) Jahrom Iran; ^2^ Deputy of Education, Department of Dermatology Jahrom University of Medical Sciences Jahrom Iran; ^3^ Department of Pharmaceutics, Faculty of Pharmacy Shiraz University of Medical Sciences Shiraz Iran

**Keywords:** case report, combination treatment, cutaneous leishmaniasis, topical therapy

## Abstract

Cutaneous leishmaniasis (CL) remains a therapeutic challenge in endemic regions, with 600,000–1 million new cases annually facing limited treatment options. We present a case series evaluating an innovative topical combination therapy for acute‐phase CL in Shiraz, Iran. Three treatment‐naïve patients (45‐year‐old female with forearm lesions, 18‐year‐old male with foot involvement, and 52‐year‐old female with hand lesions) received daily applications of a novel solution containing ciprofloxacin, ketoconazole, and metronidazole for 30 days, followed by alpha hydroxy acid for scar rehabilitation. Remarkably, all patients demonstrated complete lesion resolution and significant cosmetic improvement without treatment‐related adverse events. This well‐tolerated regimen offers several advantages over conventional systemic antimonials, including reduced toxicity, improved accessibility, and simplified administration. Our findings support the growing body of evidence for topical CL treatments, offering a potentially safer, more accessible option that warrants further clinical investigation in endemic regions. This study highlights the importance of developing targeted therapies that consider both clinical efficacy and practical implementation challenges in resource‐limited settings.

**Summary**

- This case series demonstrates the efficacy of a novel topical combination therapy for acute‐phase cutaneous leishmaniasis (CL) and achieved complete lesion resolution and significant scar improvement.
- A safe, effective, and practical option for uncomplicated CL, particularly where systemic treatments are inaccessible or high‐risk.

## 1 Background

Cutaneous leishmaniasis (CL) is a parasitic infection caused by protozoa of the genus *Leishmania*, transmitted through the bite of infected female sandflies [1, 2]. As the most common form of leishmaniasis, CL primarily presents with localized skin ulcers and accounts for an estimated 600,000 to 1 million new cases annually worldwide. Recognized by the World Health Organization (WHO) as a neglected tropical disease (NTD), CL poses significant public health and socioeconomic challenges in endemic regions [3, 4].

Leishmaniasis manifests in three principal forms—cutaneous (CL), visceral (kala‐azar), and mucocutaneous, with CL further subdivided into Old World (OWCL) and New World (NWCL) variants based on geographic distribution [5]. NWCL, endemic to the Western Hemisphere, is predominantly caused by *Leishmania braziliensis* and *L. mexicana* complexes, whereas OWCL, found in the Eastern Hemisphere, is attributed to *L. donovani* , *L. major* , *L. infantum*, *L. aethiopica* , and *L. tropica* complexes.

Historically, leishmaniasis has been regarded as a heterogeneous disease due to the wide variability in causative species and clinical presentations. Treatment strategies are tailored according to the infecting subspecies and disease severity. Systemic therapies, such as miltefosine, amphotericin B, and pentavalent antimonials (e.g., sodium stibogluconate and meglumine antimonate), remain first‐line options despite their associated toxicity. Alternatively, localized treatments, including photodynamic therapy, intralesional antimony, cryosurgery, and topical buparvaquone gel, have emerged as promising options, though evidence supporting their efficacy and safety remains limited [4, 6].

In this report, we present three clinically diagnosed CL cases from an endemic region, successfully managed with a novel liquid topical combination therapy.

## 2 Case Presentation

We report three cases of CL diagnosed clinically in Shiraz, Iran—an endemic region—all successfully treated with our novel liquid topical combination therapy during their acute disease phase. Importantly, none of the patients had received any prior treatment for their CL lesions or were taking any medications for other conditions at the time of presentation. Additionally, all patients were otherwise healthy with no underlying systemic diseases.

**FIGURE 1:**
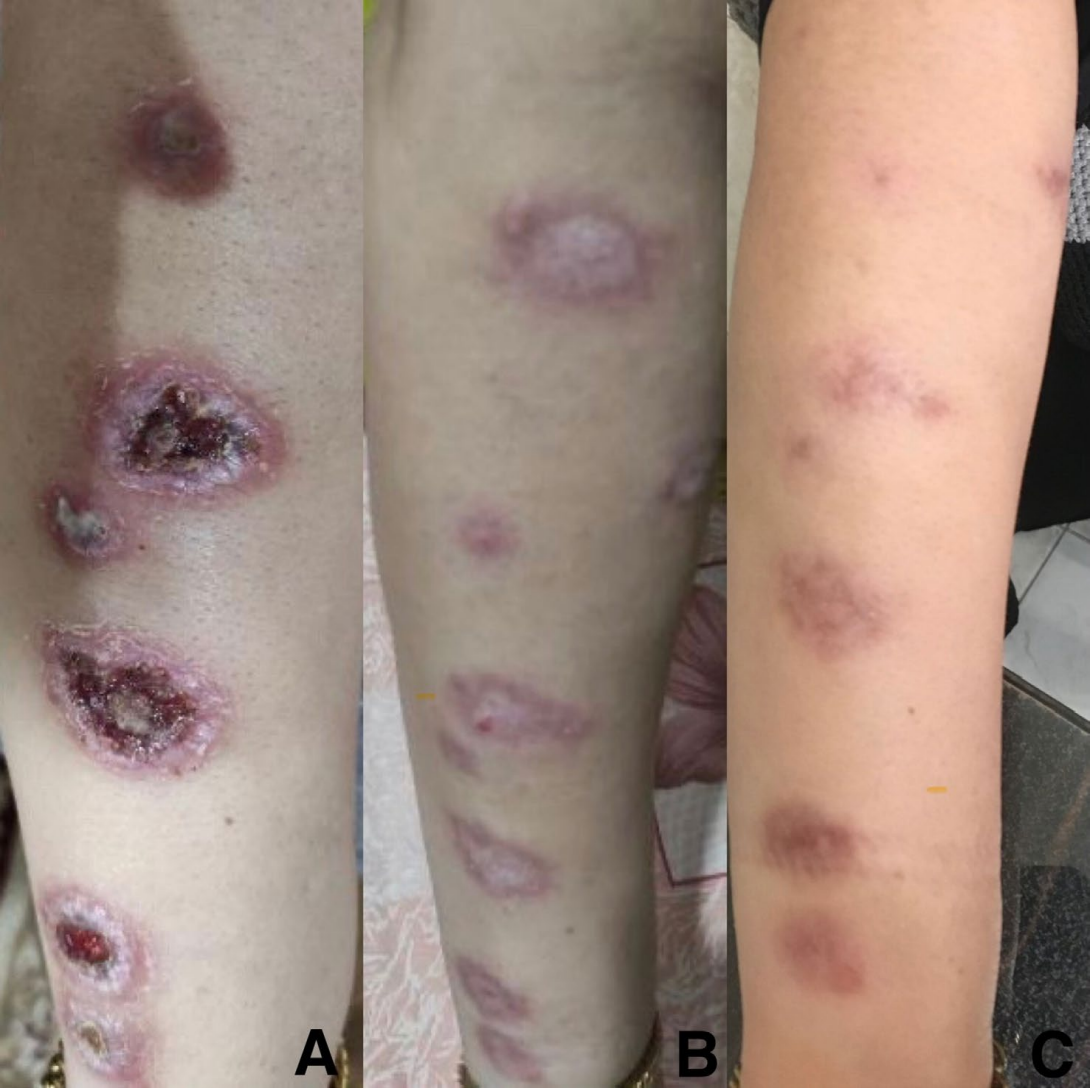
Case 1 involved multiple erythematous plaques.

**FIGURE 2:**
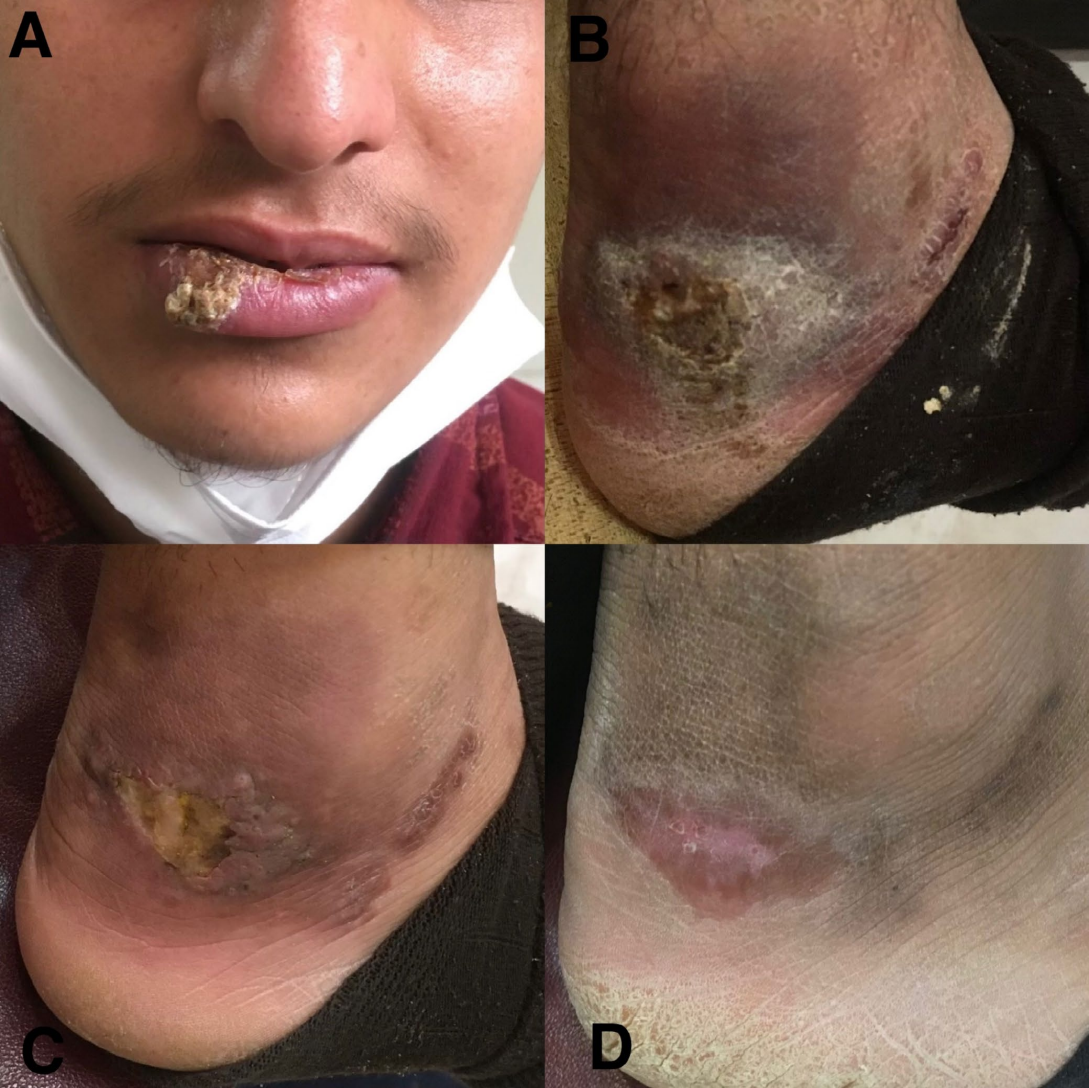
Case 2 with ulcerated erythematous plaques.

**FIGURE 3:**
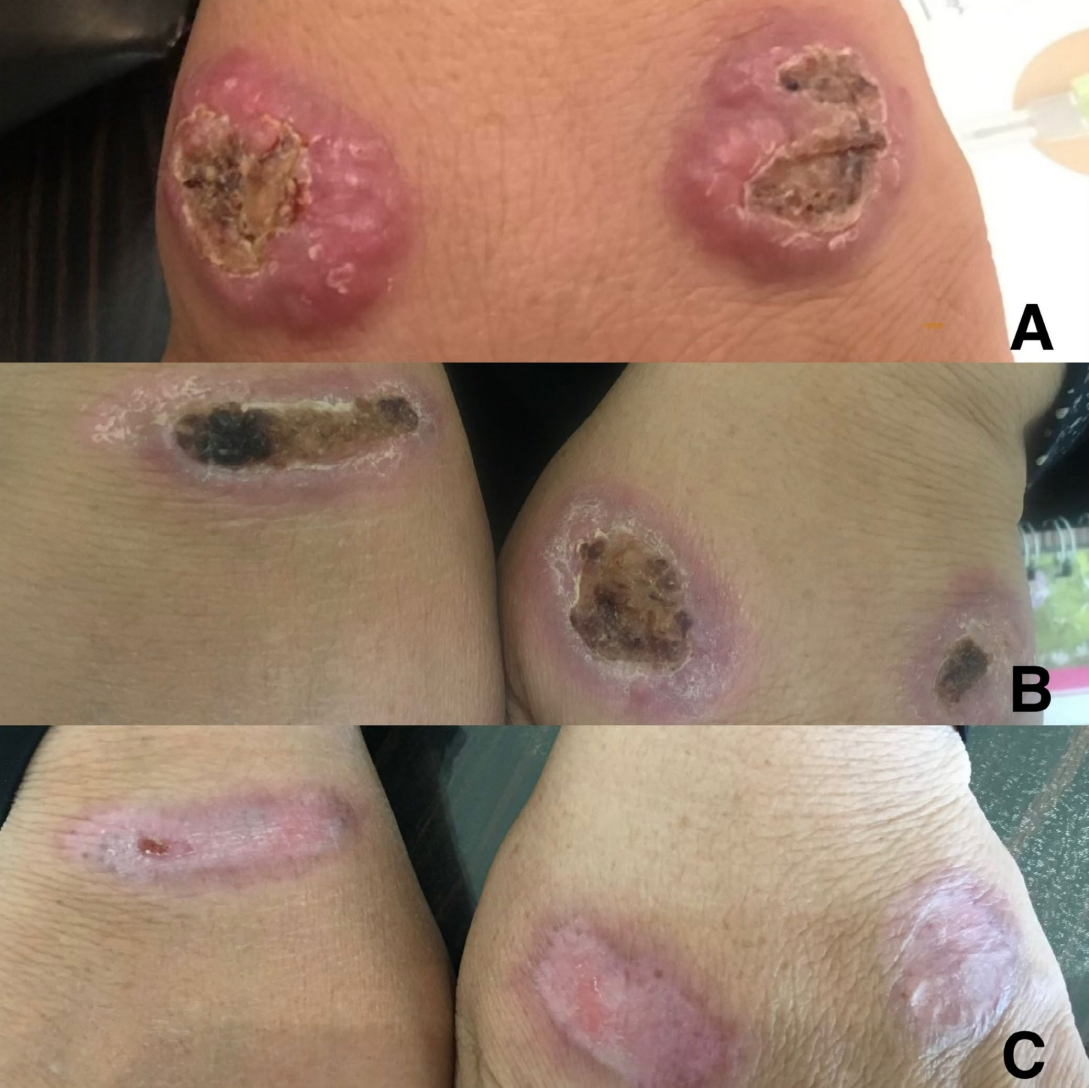
Case 3 with multiple ulcerated erythematous plaques on hands.

Case 1 involved a 45‐year‐old female presenting with multiple erythematous plaques on her right forearm showing surface crusting and ulceration (Figure 1). Case 2 was an 18‐year‐old male with a single ulcerated erythematous plaque on his right foot and her lip (Figure 2). Case 3 was a 52‐year‐old female presenting with multiple ulcerated erythematous plaques on both hands (Figure 3).

## 3 Treatment Protocol

During the acute infectious phase, all patients received our topical combination therapy consisting of:

- Ciprofloxacin (2 g).
- Ketoconazole (2 g).
- Metronidazole (2 g)suspended in 100 cc isopropyl alcohol base, applied daily for 1 month.

Following resolution of active infection, patients entered the scar management phase using 15% alpha hydroxy acid (AHA) under close monitoring for 3 months.

## 4 Treatment Outcomes

Our innovative combination therapy, initiated during the acute‐phase of disease in previously untreated patients, demonstrated excellent therapeutic results with complete lesion resolution and significant scar improvement in all three cases. The absence of comorbidities and concurrent medications in these patients provides valuable insight into the efficacy of this regimen in otherwise healthy individuals with acute CL.

## 5 Discussion

Leishmaniasis affects approximately 350 million people worldwide; the high incidence of CL makes it a significant public health concern [4]. Leishmaniasis has burdened the patient's quality of life more than our expectations for decades. Based on previous studies and reports, psychosocial and cosmetic complaints are not the only impacts on a patient's life, and other problems, such as difficulties in access to the appropriate treatments, high cost of systemic therapy, adverse reactions to the traditional usable drugs, low outcome in effectiveness, and patients' acceptance to following the routine treatments should be mentioned beside other noted impacts [1]. Whether systemic or local, treatment strategies are contingent upon the organism's subspecies; miltefosine, amphotericin B, and pentavalent antimony are commonly used for systemic therapy. In addition, local treatment has more varieties, such as photodynamic therapy, intra‐lesion antimony, cryosurgery, buparvaquone gel, and other combined solutions, which have limited data on safety and effectiveness [3, 4].

A recent systematic review by Blaizot et al. comprehensively evaluated treatment modalities for leishmaniasis across Sub‐Saharan Africa, highlighting significant regional variations in therapeutic strategies. In East Africa, Kenya predominantly employs intralesional antimonials, despite documented relapse rates associated with sodium stibogluconate, particularly in *Leishmania tropica* infections. In contrast, Sudan demonstrates clinical success with intravenous meglumine antimoniate or oral ketoconazole for both cutaneous and mucocutaneous forms. West African countries, including Senegal and Niger, preferentially administer intralesional meglumine antimoniate for *L. major* infections, supplemented by oral antifungals as secondary options. Ghana predominantly relies on traditional remedies due to limited access to conventional therapies, while Cameroon reports efficacy with topical amphotericin B deoxycholate. Southern Africa's sparse data reveal experimental use of topical paromomycin and oral fluconazole, though evidence remains limited. These findings underscore the necessity for region‐specific treatment protocols, contingent upon circulating *Leishmania* species and available healthcare infrastructure. Our liquid‐based topical treatment is a promising alternative to conventional treatments for CL. We hope our findings will contribute to developing more effective and accessible treatments for this neglected tropical disease [7].

A complementary study by Alves et al. systematically evaluated the efficacy of local amphotericin B (AmB) therapy for CL. Their comprehensive analysis revealed that various AmB formulations and administration routes have been investigated across preclinical and clinical studies, demonstrating significant advancements in therapeutic delivery technologies. The review's findings provide preliminary evidence supporting the clinical potential of locally administered AmB, suggesting it represents a promising intervention worthy of further investigation. Notably, these results establish a proof of concept for localized AmB treatment, positioning it as a viable candidate for future well‐designed clinical trials aimed at developing less toxic therapeutic alternatives for leishmaniasis management [8].

This case series demonstrates a promising, well‐tolerated treatment protocol with favorable outcomes for patients diagnosed with acute‐phase cutaneous leishmaniasis. While intralesional or parenteral antimonial administration remains the gold standard, topical therapies are emerging as viable alternatives. Our novel liquid topical combination therapy represents a significant advancement in CL treatment, offering a potentially effective alternative to conventional approaches that warrant further investigation.

## 6 Conclusion

This study demonstrates the efficacy of a novel topical combination therapy for acute‐phase cutaneous leishmaniasis, achieving complete lesion resolution and scar improvement in three treatment‐naïve patients. While antimonial therapies remain standard, our well‐tolerated liquid formulation presents a promising alternative that addresses key challenges of conventional treatments, including toxicity and accessibility. These findings support further investigation of topical approaches for CL management in endemic regions.

## Author Contributions

**Hoda Haghshenas:** supervision, validation, writing – original draft, writing – review and editing. **Aref Ghanaatpisheh:** writing – original draft, writing – review and editing. **Mohammad Darayesh:** methodology, project administration, resources, supervision, validation, writing – original draft, writing – review and editing. **Elaheh Entezar Almahdi:** writing – review and editing.

## Ethics Statement

This case report did not require ethics approval because it documents the patients' clinical course without experimental intervention. However, written informed consent was obtained from the patients and/or their legal guardians to participate in the study and publish the case details.

## Consent

The patients' parents gave their written informed consent to the publication of this case report and any accompanying images.

## Conflicts of Interest

The authors declare no conflicts of interest.

## Data Availability

All relevant data generated or analyzed during this study is included in this published article. Additional information is available from the corresponding author upon reasonable request.
